# Misophonia: Diagnostic Criteria for a New Psychiatric Disorder

**DOI:** 10.1371/journal.pone.0054706

**Published:** 2013-01-23

**Authors:** Arjan Schröder, Nienke Vulink, Damiaan Denys

**Affiliations:** 1 Department of Psychiatry, Academic Medical Center, University of Amsterdam, Amsterdam, The Netherlands; 2 The Institute for Neuroscience, Royal Netherlands Academy of Arts and Sciences, Amsterdam, The Netherlands; Institute of Psychiatry at the Federal University of Rio de Janeiro, Brazil

## Abstract

**Background:**

Some patients report a preoccupation with a specific aversive human sound that triggers impulsive aggression. This condition is relatively unknown and has hitherto never been described, although the phenomenon has anecdotally been named misophonia.

**Methodology and Principal Findings:**

42 patients who reported misophonia were recruited by our hospital website. All patients were interviewed by an experienced psychiatrist and were screened with an adapted version of the Y-BOCS, HAM-D, HAM-A, SCL-90 and SCID II. The misophonia patients shared a similar pattern of symptoms in which an auditory or visual stimulus provoked an immediate aversive physical reaction with anger, disgust and impulsive aggression. The intensity of these emotions caused subsequent obsessions with the cue, avoidance and social dysfunctioning with intense suffering. The symptoms cannot be classified in the current nosological DSM-IV TR or ICD-10 systems.

**Conclusions:**

We suggest that misophonia should be classified as a discrete psychiatric disorder. Diagnostic criteria could help to officially recognize the patients and the disorder, improve its identification by professional health carers, and encourage scientific research.

## Introduction

In 2009 three patients were referred to our expertise centre in obsessive-compulsive disorders (OCD) at the Academic Medical Center in Amsterdam with obsessions focused on a typical sound such as smacking or breathing and the subsequent aggressive impulse to scream and yell or attack the source of the sound in order to make it stop.

This cluster of symptoms does not fit any of the well-known obsessive-compulsive or impulse control disorders, but has been anecdotally referred to as misophonia, meaning hatred of sound [1]. As of yet, two case reports have been published in the field of audiology and psychosomatic medicine, although several misophonia newsgroups and networks (e.g. http://www.misophonia-uk.org) indicate that this condition may occur more often than is currently assumed [2], [3]. The suspicion of a wider spread prevalence of this disorder was supported by the number of patients with misophonia that were (self-) referred to our hospital following an announcement on a Dutch misophonia Internet newsgroup and our hospital website. Within 2.5 years, nearly 50 misophonia patients contacted our hospital.

The symptoms, personality traits and coping mechanisms of the patients showed a striking similarity in nature and development. The consistent pattern of symptoms suggested the presence of a discrete and independent disorder. However, within the current classification systems, the Diagnostic and Statistical Manual of Mental Disorders, Fourth Edition, Text Revision (DSM-IV-TR) [4] and the International Statistical Classification of Diseases and Related Health Problems, 10^th^ revision (ICD-10) [5], there is no option to officially classify the disorder. In this paper we describe the clinical symptomatology of misophonia, discuss the classification of symptoms, propose diagnostic criteria for misophonia, and introduce a concept assessment scale, the A-MISO-S.

## Methods

42 Dutch patients were clinically assessed using a standard psychiatric interview by five psychiatrists experienced in obsessive-compulsive spectrum disorders. The general medical history as well as the psychiatric history was collected for all patients. Personality pathology was evaluated using the Structured Clinical Interview for DSM-IV Axis II Personality Disorders (SCID-II) [6]. The following questionnaires were completed:

The Hamilton Depression Rating Scale (HAM-D) [7], a 17-item scale determining a patient’s level of depression.

The 14-item Hamilton Anxiety Rating Scale (HAM-A) [8], which measures the severity of anxiety symptoms.

The Symptom Checklist (SCL-90) [9], which is a widely used screening instrument for mental and physical dysfunctioning. The 90 items comprise eight subscales: Agoraphobia, Anxiety, Depression, Somatic complaints, Insufficiency in thinking and acting, Suspicion and interpersonal sensitivity, Hostility and Sleep problems. The total score is seen as a general index for psychoneuroticism.

To measure the severity of the misophonia symptoms, we developed an adapted version of the Yale-Brown Obsessive-Compulsive Scale (Y-BOCS) [10], [11], which we have named the Amsterdam Misophonia Scale (A-MISO-S). Similar adaptations of the Y-BOCS have appeared to be reliable and valid measures of symptom severity in other obsessive-compulsive and impulse control disorders, such as pathological gambling (PG-YBOCS) [12] and body dysmorphic disorder (BDD-YBOCS) [13].

On a 6-item scale (range 0–24) patients were asked about the (1) time they spent on misophonia; (2) interference with social functioning; (3) level of anger; (4) resistance against the impulse; (5) control they had over their thoughts and anger; and (6) time they spent avoiding misophonic situations. Scores from 0–4 are considered subclinical misophonic symptoms, 5–9 mild, 10–14 moderate, 15–19 severe, 20–24 extreme.

To rule out any potential hearing problems we randomly selected five patients to perform a standard hearing test, including pure tone, speech audiometry and loudness discomfort levels, which are commonly performed to objectify hearing loss or distortion [14], [15]. One patient’s test showed unexplained conductive hearing loss. In the other four patients no significant audiological distortion was found and further testing was therefore omitted.

The medical ethics testing committee of the Academic Medical Center did not require approval because this study was anecdotal and observational. All patients gave written informed consent for publication.

## Results

Table 1 provides the demographic and clinical characteristics of the patients. 52% were males and the mean age of onset was 13 years (range 2–38). In all 42 patients we found a remarkably similar pattern of symptoms: 1. Triggering stimuli were all sounds produced by humans. Animal or other sounds usually did not cause distress, nor did sounds made by the patients themselves. Symptoms in 34 patients (81%) were triggered by eating-related sounds like lip smacking. 27 patients (64.3%) mentioned (loud) breathing or nose sounds as provocative. 25 patients (59.5%) could not tolerate the sound of typing on a keyboard or pen clicking sounds. 2. The stimuli were initially auditory and sometimes expanded to visual stimuli, with the image directly related to the triggering sound (e.g. watching someone else eat also caused arousal). Five patients (11.9%) reported a misophonia-like reaction when confronted with certain repetitive visual movements made by another person such as leg rocking (in analogy to misophonia this can be named *misokinesia*, meaning hatred of movement). 3. Exposure to the misophonic stimulus provoked an immediate aversive physical reaction, starting with irritation (59.5%) or disgust (40.5%) that instantaneously became anger. 12 patients (28.6%) described getting verbally aggressive on occasions. Seven patients (16.7%) admitted physical aggression directed towards objects. Five patients (11.9%) hit an (ex-) partner once. Anxiety was explicitly not experienced. 4. The intensity of the anger with rare but potential aggressive outbursts initiated a profound feeling of loss of self-control. 5. Patients had insight and perceived their aggressive reaction as excessive and unreasonable and estimated the loss of self-control as morally unacceptable. 6. All patients actively avoided the misophonic stimuli by avoiding social situations, wearing headsets or producing anti-sounds that resulted in marginal social contacts. 7. Patients experienced daily stress or discomfort by anticipating an unexpected encounter with misophonic stimuli. The severity of symptoms on the concept A-MISO-S was severe (15.1 out of 24. Range: 9–22). Three patients (7.1%) were diagnosed with a comorbid mood disorder. Depressive and anxiety symptoms and overall psychoneuroticism were reported higher than in the general population (HAM-D score: mean 7.3, range 0–22; HAM-A: mean 11.2, range 0–31; SCL90: mean 156.7, range 93–294). 8. Their personality showed traits of obsessive-compulsive personality disorder (OCPD). 22 patients (52.4%) met the criteria for OCPD.

**Table 1:** Demographic and clinical characteristics of the misophonia patients (N = 42).

Sex (%) (N)	Male 52.4 (22)
Age of onset (mean – range)	13 (2–38)
Age at diagnosis (mean – range)	37 (19–62)
Triggering sounds (N – (%))	
Eating sounds^1^	34 (81)
Breathing/nose sounds^2^	27 (64.3)
Finger/hand sounds^3^	25 (59.5)
Foot sounds^4^	7 (16.7)
Repetitive visual movements^5^	5 (11.9)
Aggressive reaction (N – (%))	
No aggression	17 (40.5)
Verbal aggression	12 (28.6)
Directed towards objects	7 (16.7)
Physical aggression in the past	5 (11.9)
Occasional physical aggression	1 (2.4)
Psychiatric comorbidity (N – (%))	
Mood Disorder 6	3 (7.1)
Panic disorder	1 (2.4)
ADHD^7^	2 (4.8)
Tourette Syndrome	2 (4.8)
Hypochondria	1 (2.4)
OCD^8^	1 (2.4)
OCPD9, a	22 (52.4)
TTM^10^	2 (4.8)
Skinpicking	1 (2.4%)

1 (Lip) smacking, swallowing, eating chips/fruit.

2 Loud breathing, nostril sounds, coughing, sneezing.

3 Typing, pen clicking, nail clipping.

4 Footsteps, sound of high heels.

5 Repetitive leg rocking.

6 (Recurrent) depressive disorder, dysthymic disorder.

7 Attention Deficit Hyperactivity Disorder.

8 Obsessive Compulsive Disorder.

9 Obsessive Compulsive Personality Disorder.

10 Trichotillomania.

a No other personality disorders were diagnosed in the sample.

## Discussion

In the past three years we meticulously screened 42 patients with symptoms of misophonia, a condition that has not been described yet in the psychiatric literature. This sample represents, to our knowledge, the largest group described worldwide. We found in all patients a similar pattern of intense anger when hearing certain human sounds, impulsive reactions, avoidance of cue-related situations, worry of losing control, and the occurrence of obsessive compulsive personality traits.

Although misophonia is completely absent in the psychiatric literature, two case reports have been described in audiology and psychosomatic medicine [2], [3]. These cases have a similar age of onset and a comparable pattern of symptoms with avoidant behaviour resulting in social dysfunctioning. A minor difference with our sample, however, is the presence of distress instead of anger in two of these cases.

Aversive reactions, i.e. discomfort that is not fear or anxiety, following various stimuli have also been described by Marks [16]. Most commonly, these are revulsions of touching specific materials like cotton wool or velvet and taste or smell aversions that cause people to avoid certain foods. Notable auditory aversions are to scraping sounds, like chalk on a blackboard. The major difference with our sample is that the four misophonic categories, such as eating related stimuli (table 1), are not mentioned.

The symptom pattern of misophonia shares a number of features with other DSM-IV-TR and ICD-10 diagnoses: specific phobia, post-traumatic stress disorder (PTSD), social phobia, obsessive compulsive disorder (OCD), intermittent explosive disorder, emotionally unstable personality disorder, borderline personality disorder, antisocial personality disorder, OCPD, and autism spectrum disorders (ASD). It also shares similarities with sensory processing disorders (SPD) and phonophobia. Even though misophonia resembles these disorders, none of the diagnostic categories fit the whole symptom pattern of misophonia:

### (i) Specific Phobia

Specific phobia resembles misophonia because in specific phobia an external acoustic stimulus can also trigger a negative emotional reaction when this stimulus is directly related to the phobic object (i.e. the sound of a dog barking in fear of dogs). As in misophonia, this emotional reaction causes avoidant behaviour. However, in specific phobia the perceived emotion is anxiety, whereas in misophonia it is aggression.

### (ii) PTSD

As in misophonia and specific phobia, in PTSD acoustic stimuli can cause intense aversive physical arousal with subsequent avoidant behaviour. PTSD-related stimuli can arouse a sudden recollection and/or re-enactment of the trauma, or of the original reaction to it, and may even trigger dramatic, acute bursts of fear, panic or aggression. However, in PTSD a life threatening traumatic event has been experienced and the driving emotion is fear, not aggression.

### (iii) Social Phobia

Both patients with social phobia and misophonia experience stress or anxiety in social situations and will avoid these. In social phobia the core is a hypersensitivity to negative evaluation by others. However, in misophonia the fear of social situations is secondary to concerns of encountering misophonic stimuli.

### (iv) OCD

In misophonia there is a monothematic preoccupation with a specific sound, which resembles obsessionality in OCD. Avoidance is present in both disorders as well as in other anxiety disorders. However, in OCD patients tend to perform compulsive acts to reduce anxiety and aggression is not commonly reported.

### (v) Intermittent Explosive Disorder

Excessive impulsive aggression is seen in various disorders, most notably in intermittent explosive disorder [17]. In this condition there must be several separate episodes of failure to restrain aggressive impulses that result in serious assaults against others or property destruction. Patients with misophonia also report impulsive aggression but very rarely lose control for they feel it is unacceptable and that it should be prevented at all costs.

### (vi) Personality Disorders with Impulsive Aggression

In various personality disorders impulsive aggression is defined. In emotionally unstable personality disorder (ICD-10), borderline personality disorder (DSM-IV-TR) and antisocial personality disorder (DSM-IV-TR, ICD-10) there is frequent impulsivity and difficulty controlling anger but this is not related to any specific sound. However, in misophonia aggressive outbursts are rare. Moreover, none of the patients meet the criteria for these personality disorders.

### (vii) OCPD

People with misophonia show characteristics of OCPD (or anankastic personality disorder, ICD-10). Twenty-two patients (52.4%) even meet the SCID-II criteria for OCPD. However, not all OCPD patients report misophonic symptoms. Furthermore, aggression is not mentioned as a key symptom of this disorder. This high comorbidity does raise the question of whether OCPD is a predisposing factor in the development of misophonia or a consequence of having misophonia. It has been reported previously that some individuals with impulsive aggressive problems develop OCPD symptoms in an attempt to compensate for an underlying problem with behavioural inhibition [18]. This does not hold for our sample because in misophonia the impulsive aggression is only related to certain sounds. Also, none of our patients noticed a direct association between their misophonia and personality. They suffered most from misophonia, whereas OCPD did not cause them much suffering.

### (viii) ASD and SPD

Auditory hyper-responsivity is also observed in ASD. It is even thought that a dysfunction in different sensory modalities is characteristic for ASD [19]. Regarding this sensory dysfunction in ASD there is an overlap with the concept of SPD, a group of disorders that involve challenges in modulation, integration, organization, and discrimination of sensory input that causes inadequate responses to the input and disruptive emotional and behavioural patterns [20]. Typical auditory sensitivity in ASD and SPD is to unexpected and loud noises, such as vacuum cleaners or a dog barking [21]. This pattern is clearly different from the auditory triggers in the misophonia patients. Furthermore, none of our patients was diagnosed with ASD. Since the validity of SPD is still not widely accepted, further research is needed on this concept.

### (ix) General Medical Conditions and Substance Induced Disorders with Impulsive Aggression

Aggression can be related to the consumption of intoxicating substances. However, none of the patients used recreational drugs or excessive amounts of alcohol, so these can be ruled out as a cause. Moreover, none of the patients reported any physical conditions in general or history of brain damage in particular. Although one patient showed hearing loss in the right ear, it is unlikely that an organic cause could explain the symptoms.

### (x) Phonophobia

A strong emotional reaction to sound is also described in phonophobia (i.e. fear of sound). It has been suggested that phonophobia is an extreme form of misophonia [22]. However, if that were the case, the patients would experience anxiety at some stage in the course of their condition. Our patients were therefore explicitly asked about the occurrence of anxiety in relation to the misophonic stimuli. None of them reported experiencing anxiety, therefore this was excluded.

### Aetiology of Misophonia

It is, as of yet, difficult to hypothesize about the aetiology and course of misophonia. Patients themselves often report that the onset of the disorder is associated with a profound disgust of hearing family members eating during childhood. One can imagine a process of recurrent conditioning following these repetitive annoying events that eventually results in misophonic symptoms or avoidant behaviour [23]. Though these events may have triggered the disorder, all patients deny traumatization or the experience of trauma-related events. Another explanation is that misophonia is part of a general hyper-reactivity syndrome to sensory stimuli. This might explain the shift from auditory to visual stimuli. If so, misophonia should share similarities with the concept of SPD, which is not the case. Another hypothesis is that OCPD predisposes to misophonia. A morally strict person may have difficulties coping with impolite eating sounds, eventually resulting in avoidance. Conversely, as stated earlier, OCPD symptoms can develop in impulsive aggressive patients, as a compensation for an underlying problem with behavioural inhibition [18]. From a phenomenological viewpoint, there appears to be an obsessional part, the focus and preoccupation on a particular sound, and an impulsive part, the urge to perform an aggressive action. Both aspects should optimally be explained within one single causal model, which currently is too ambitious.

### Diagnostic Criteria

Since the symptomatology of misophonia does not fit into DSM-IV-TR or ICD-10 classifications, we suggest a unique set of diagnostic criteria based on the pattern of symptoms observed in our patients (Table 2). We suggest that misophonia should be considered as an obsessive compulsive spectrum disorder (OCSD) [24]. The OCSDs have in common symptoms of obsessionality, compulsivity or impulsivity. They also share similarities in the course of illness, patient population, treatment response and neurocircuitry. In our patient cohort we observed comorbid OCSDs such as Attention Deficit Hyperactivity Disorder (two patients), hypochondria (one), Tourette syndrome (two), OCD (one), OCPD (twenty-two), skinpicking (one) and trichotillomania (two). Speculatively, if misophonia were to be considered within the OCSDs, it could be classified on the impulsivity pole of the spectrum. The validity of OCSDs is still under debate and classifying misophonia as such should be considered premature.

**Table 2:** Proposed diagnostic criteria for misophonia.

A.The presence or anticipation of a specific sound, produced by a human being (e.g. eating sounds, breathing sounds), provokes an impulsive aversive physical reaction which starts with irritation or disgust that instantaneously becomes anger.
B.This anger initiates a profound sense of loss of self-control with rare but potentially aggressive outbursts.
C.The person recognizes that the anger or disgust is excessive, unreasonable, or out of proportion to the circumstances or the provoking stressor.
D.The individual tends to avoid the misophonic situation, or if he/she does not avoid it, endures encounters with the misophonic sound situation with intense discomfort, anger or disgust.
E.The individual’s anger, disgust or avoidance causes significant distress (i.e. it bothers the person that he or she has the anger or disgust) or significant interference in the person’s day-to-day life. For example, the anger or disgust may make it difficult for the person to perform important tasks at work, meet new friends, attend classes, or interact with others.
F.The person’s anger, disgust, and avoidance are not better explained by another disorder, such as obsessive-compulsive disorder (e.g. disgust in someone with an obsession about contamination) or post-traumatic stress disorder (e.g. avoidance of stimuli associated with a trauma related to threatened death, serious injury or threat to the physical integrity of self or others).

### The Amsterdam Misophonia Scale (A-MISO-S)

The A-MISO-S is a concept scale based on the Y-BOCS that was used to assess the patients. The A-MISO-S questionnaire is available in Figure S1.

### Conclusion

In the present study we investigated 42 patients with misophonia. A specific acoustic cue, produced by a human being, provoked an impulsive aversive physical reaction with irritability, disgust and anger. The intensity of these emotions provoked a fear of uncontrollability with subsequent avoidance which was evaluated with the concept A-MISO-S. Patients showed aspects of OCPD. Hearing tests did not reveal any underlying deficits. Misophonia cannot be classified under current disorders within DSM-IV-TR and ICD-10 and should be delineated as a separate psychiatric disorder. We propose diagnostic criteria which could improve recognition by health carers and encourage scientific research.

## Supporting Information

Figure S1 **Amsterdam Misophonia Scale (A-MISO-S).**
(TIFF)
