# Supplementary figures and images for: Misophonia: Diagnostic Criteria for a New Psychiatric Disorder
Source: PLoS One. 2013 Jan 23;8(1):e54706. doi: 10.1371/journal.pone.0054706 (PMC3553052; doi:10.1371/journal.pone.0054706)

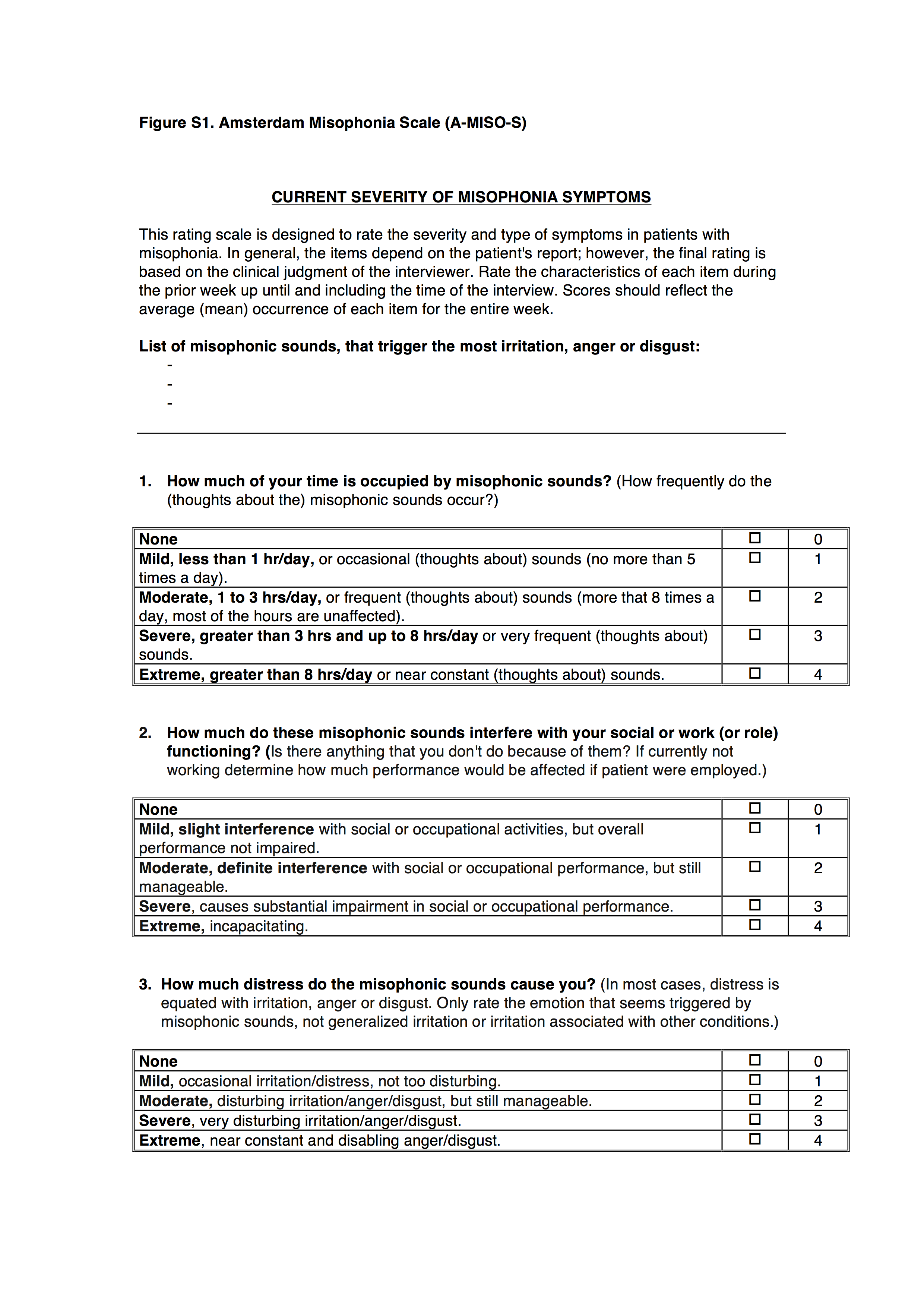

Supplement: Figure S1 — Amsterdam Misophonia Scale (A-MISO-S). (TIFF) [file pone.0054706.s001.tiff]
